# Allergic diseases in infancy: I - Epidemiology and current interpretation

**DOI:** 10.1016/j.waojou.2021.100591

**Published:** 2021-11-12

**Authors:** Isabella Annesi-Maesano, Manja Fleddermann, Mathias Hornef, Erika von Mutius, Oliver Pabst, Monika Schaubeck, Alessandro Fiocchi

**Affiliations:** a Institute Desbrest of Epidemiology and Public Health, Montpellier University and University of Montpellie, France; b HiPP GmbH & Co. Vertrieb KG, Georg-Hipp-Straße 7, Pfaffenhofen, 85276, Germany; c Institute of Medical Microbiology, RWTH Aachen University Hospital, Pauwelsstr. 30, Aachen, 52074, Germany; d Dr. von Hauner Children's Hospital, University of Munich, Lindwurmstr. 4, Munich, 80337, Germany; e Institute of Molecular Medicine, RWTH Aachen University, Pauwelsstr. 30, Aachen, 52074, Germany; f Division of Allergy, Pediatric Hospital Bambino Gesú (IRCCS), Piazza di Sant’Onofrio 4, Rome, 00165, Italy

**Keywords:** Allergy, Allergy prevention, Farm effect, Hygiene hypothesis

## Abstract

**Objective:**

Among non-communicable diseases, the prevalence of allergic diseases has increased significantly in the new millennium. The increase of allergic diseases is linked to the changing environment of infants.

**Methods:**

This narrative review summarizes the discussions and conclusions from the 8^th^ Human Milk Workshop. Information from the fields of pediatrics, epidemiology, biology, microbiology, and immunology are summarized to establish a framework describing potential avenues for the prevention of allergic diseases in the future.

**Results:**

Several environmental circumstances are linked to the development of allergic diseases. While cesarean section is increasing the risk of allergies, early childhood exposure to a farm environment has a protective effect. From their analysis, nutritive and non-nutritive factors influencing the allergy risk in later life have been identified. The effect of breastfeeding on food allergy development is non-univocal. Human milk components including immunoglobulins, cytokines, and prebiotics have been indicated as important for allergy prevention.

**Conclusion:**

Many factors linked to the western lifestyle have been associated with the development of allergic diseases. This suggests several theories that may serve as a basis for new protective interventions. While it is indubitable that mother's milk protects from infectious diseases, its role in the prevention of allergic diseases is to be elucidated.

## Introduction

Food allergy has increased significantly for reasons that remain unknown. In 2011, alarming data from Australia pointed to a rise in food allergies in infants and children. This highlighted the need to identify the causes of this phenomenon and to identify appropriate preventive strategies.^1^ Now, 10 years later, we know far more. However, it is still difficult to translate this knowledge into effective preventive strategies, as shown by the changing recommendation guidelines of international scientific societies. These societies are still skeptical of the results achieved so far.^2^^,^^3^

This narrative review is based on a workshop, which discussed questions of allergy epidemiology and causality in order to transfer current research findings into practice.

### Food allergies are increasing worldwide

The prevalence of food allergies is increasing around the globe posing a significant public health issue.^4^ In industrialized countries, food allergy is the leading cause of anaphylaxis seen in emergency departments. In developing countries, however, concerns are rising about managing and preventing complications.^5^^,^^6^

While the prevalence of food allergies in children born in the United Kingdom (UK) between 2005 and 2007 was about 3.5%, the prevalence in infants born between 2009 and 2012 was already twice as high (7.1–7.3%).^7^ Data from Asian countries show a similar development.^8^, ^9^, ^10^

In China, the prevalence of food allergies proved by an oral food challenge amongst 0 to 24 months-old infants increased from 3.5% to 7.7% between 1990 and 2009.^11^

The global increase in food allergy prevalence can be attributed in particular to a few specific allergies such as egg allergy. The EAT study conducted in the United Kingdom found an egg allergy prevalence of 5.5%, compared to 2.2% found by EuroPrevall.^12^ The prevalence of challenge-confirmed egg allergy varies between 0.11% in Greece and 1.95% in the United Kingdom.^12^ In Asia, the prevalence of egg allergy is 2 times higher than that of milk allergy.^13^ Australian data show an egg allergy prevalence of 8.9% in the first 2 years of life.^14^

On the other hand, milk allergy rates appear to remain constant. The incidence in European children born between 2005 and 2007 was about 0.8% and the same rate was observed in children born between 2009 and 2010.^15^

Milk allergy prevalence varies between 0.23% in Lithuania and 1.26% in the United Kingdom.^15^ Asian data collected in a birth cohort from Singapore confirms that milk allergy accounts for only a small proportion of food allergies.^13^

Atopic diseases are becoming an epidemic and pandemic issue. Projections suggest that the global population is likely to reach 9 to 10 billion people by 2050, with 2 to 4 billion of them suffering from allergic diseases, such as asthma, allergic rhinitis, and atopic dermatitis.^16^ Therefore, it is important to find new and effective ways to prevent asthma and other atopic diseases. This is only possible if we understand the connection between the development of atopic diseases, the host, and the environmental factors involved.

### The exposome and its role in allergy development

Environmental factors have a huge impact in the development of food allergies. At the population level, these factors play a major role in the development of all non-communicable diseases. They are summarized under the term exposome, which is defined as “the totality of specific and nonspecific external environmental exposures […] to which a subject is exposed from preconception onward and their consequences at the organ and cell levels”.^17^

**Fig. 1:**
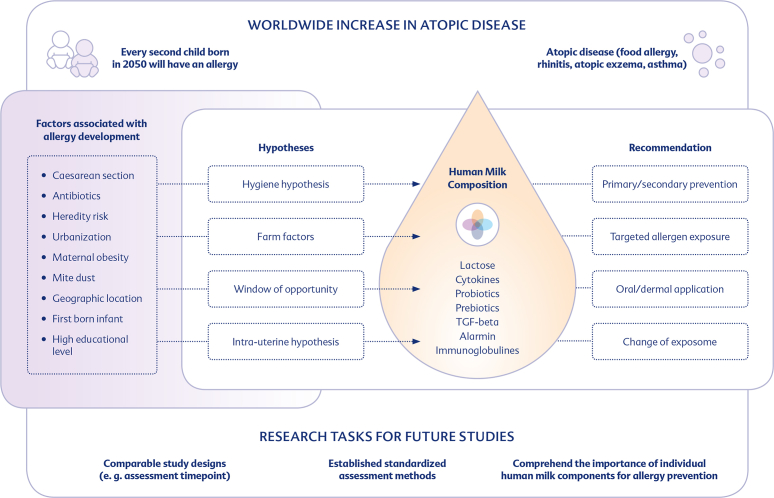
Current knowledge regarding the factors associated with allergy development and its underlying hypotheses, as well as the allergy-preventive components in human milk and current recommendations and tasks to enable allergy prevention

The increase in atopic diseases reflects the changed social and environmental conditions in which the world population lives.^18^ There are different hypotheses that seek to explain the increase in atopic diseases (see Fig. 1).

#### The impact of ethnicity and genetics on the development of food allergies is low

Ethnicity may play a role in allergy development, but is assumed to be of minor relevance. In Australia, children born to mothers of Asian descent have an increased risk of positive nut antigen testing at school age compared to children born to mothers of Caucasian descent.^19^ A meta-analysis focusing on genetic markers for the development of allergic and respiratory diseases suggests that the genes associated with asthma are linked to airway inflammation, total IgE levels, as well as the immune response to viruses within a genetically homogeneous population. Associations in children of multi-ancestry origin are weaker compared to homogeneous populations.^20^

Several genetic variables have also been associated with the development of atopic dermatitis and food allergies: Filaggrin (FLG) is an important protein for the aggregation of filaments within the epidermis. Loss-of-function FLG mutations have been linked to peanut allergy, but the correlation is weak.^21^ Also, findings for the association between the human leukocyte antigens (HLA) and food allergies are inconsistent. No polymorphism in the HLA region has been found to reach a genome-wide significance level for food allergy development.^22^ Two polymorphisms were associated with peanut allergy, but only in children of European ancestry.^23^ No variations were found with respect to milk and egg allergy.^24^ It therefore seems that the linkage disequilibrium in HLA genes could play a role in persistent food allergies rather than in those with a more favorable natural history that terminate in early childhood.

In conclusion, these data suggest that genetic factors are important on food allergy development, but their expression is filtered through environmental factors.^25^ Adequately powered studies, targeted to specific clinical conditions, will be necessary to confirm the value of these associations.

#### Socioeconomic and geographical aspects

Looking at the world allergy map, the most affected regions with high incidence rates are Europe, North America, South America, and Australia. Hence, countries with a high gross net income per capita have the highest allergy rates.^26^ A study using the GINI index (an economic index indicating the equality of income distribution in a country) suggests a correlation between economic inequality and asthma prevalence.^27^ This means that allergies seem to be diseases of developed countries.^18^

A higher parental educational level is associated with an increased risk of allergies.^18^ With one or both parents having a college degree, the probability of infants becoming allergic is doubled.^28^ The educational level of parents is closely related to factors known to influence allergy risk such as number of siblings and mode of delivery.^28^^,^^29^ Having a sibling reduces the allergy risk by 50%.^28^ This also means that the first-born infant is 2 times more likely to develop an allergy than the fourth-born sibling.^18^

An urban environment is another factor that increases the risk of allergies. Living in a smaller city or village, on the other hand, seems to protect against allergies.^6^ Living in neighborhoods with little green space, or to soil/soil bacteria can contribute to the development of food allergies.^30^ Further possible contributors to a higher allergy risk in urban areas could be air pollution (eg, diesel fuel, ozone) as well as chemical contaminants in water, soil, or packaged foods. The exposure to these pollutants is associated with allergy development.^31^

#### Allergen and microbial exposure may protect from allergic diseases

Environmental factors play an important role in modulating the microbiota profile during the sensitive period of infancy and thus also in the development of allergies. Several of these factors have been identified in epidemiological studies of allergy development. The hygiene hypothesis postulates that a reduced exposure to allergens and microbial products in early infancy and childhood shifts T-helper cells to a predominant Th2-skewing, thereby creating a predisposition for allergic diseases.^30^

The intestinal microbial ecosystem seems to be linked to the risk of allergy development. In a cross-sectional study, the microbiota profile of children of Chinese origin, born either in continental China or in Western Australia, showed significant differences in alpha-diversity. Chinese children in Western Australia have a significantly less diverse microbiota due to their Western environment, and this was related to the development of food allergies and wheezing.^32^

Cesarean section, the use of antibiotics early in life, or extreme dietary habits change the infant's microbiota and have been linked to an increased incidence and severity of allergic diseases.^33^ Antibiotic prophylaxis, used routinely for cesarean section, increased the risk of food allergies in infants during the first 3 years of life.^33^^,^^34^

In conclusion, a wide-ranging exposure to allergens and microbial products during pregnancy or infancy may protect against allergies.

#### Maternal factors and nutrition linked to allergy development

The intra-uterine hypothesis associates prenatal factors — such as maternal obesity, the number of pregnancies, and the use of antibiotics — with the maternal immune status, which in turn has an impact on the infant's risk of allergies.^35^ For example, maternal obesity may be linked to atopic dermatitis.^36^ Furthermore, maternal stress during pregnancy might be involved in allergy development.^37^

The exposure to allergens such as pollen or food allergens during pregnancy alters the infant's immune development and might therefore be related to allergy development in the offspring.^38^ Furthermore, numerous nutritional factors such as dietary antioxidants, lipids, and vitamins during pregnancy have been linked to the infant's risk of atopic dermatitis, allergic sensitization, and asthma.^39^

Given that the allergy risk highly depends on geographical, socioeconomic, and hereditary factors, each region/country has its own priorities regarding allergy prevention strategies that focus on dietary recommendations. Only few recommendations are applicable worldwide, eg, the recommendation to solely breastfeed during the first 4 to 6 months after birth.^40^^,^^41^ Currently there are no global allergy prevention guidelines focusing on nutrition for the pre-conception, pregnancy, or lactation periods.

The use of probiotics during pregnancy and infancy might reduce the risk of atopic dermatitis and other allergic diseases. While it is recommended by a World Allergy Organization (WAO) panel, national guidelines have not yet adopted any such recommendation.^42^^,^^43^

After 4 to 6 months of exclusive breastfeeding, recommendations suggest the introduction of complementary foods into the infant's diet,^40^^,^^41^ which exposes them to potentially allergenic dietary antigens. Some food components that promote the formation of advanced glycation end products, which is supported by high sugar intake (eg, from the consumption of fast food), have been associated with the development of allergies and other chronic diseases such as diabetes or obesity.^44^

### Human milk research: the key to allergy prevention

Human milk is the natural food for infants during a sensitive period of immune and metabolic programming and provides unique nutritional benefits. A meta-analysis on breastfeeding and its life-long effects concluded that breastfeeding has a beneficial effect on many aspects of human health.^45^ The meta-analysis included data from both low- and middle-income countries.

Human milk consists of proteins, fats, carbohydrates (mainly lactose), oligosaccharides, vitamins, minerals, and a huge number of protective immune-modulating substances. These components are present in the milk of all species, but their specific types and proportional amounts vary between species and individuals, as well as throughout the lactation period: The colostrum (birth until 4 days) is rich in protein, fat-soluble vitamins, minerals, and immunoglobulins compared to the transitional and mature milk.^46^

A plethora of beneficial effects is attributed to single components of human milk. Human milk contains a total of 268 proteins, which release bioactive peptides, which are important for the modulation of the immune system.^47^ Prebiotics, like human milk oligosaccharides (HMOs), are non-digestible carbohydrates that positively modulate the infant's microbiota through their bifidogenic effect.^48^ Cytokines such as interleukin (IL)-4, IL-5, and IL-13 as well as transforming growth factor-beta (TGF-β) are important in allergy development. Furthermore, immunoglobulins such as secretory immunoglobulin A (sIgA) in milk reflects the mothers "immune history", thus providing protection against pathogens and shaping the developing infant microbiota.^49^^,^^50^

The composition of human milk can be modified through maternal dietary intervention, eg, consumption of fish oil or probiotic supplementation.^51^ However, whether this has an effect on clinical outcomes has to be demonstrated, yet.

#### Epidemiological evidence of breastfeeding and allergy prevention

The association among breastfeeding and food allergies is complex. While breastfeeding reduces the risk of food allergies in the general population,^52^^,^^53^ the results for high-risk children are inconsistent.^54^, ^55^, ^56^ The failure in providing consistent results is caused by varying study-setups and the lack of standardized methods, like double-blind food challenge tests, skin-prick test, or specific IgEs.

Looking at other allergic diseases such as atopic dermatitis, data from cross-sectional studies and birth cohorts indicate that breastfeeding and atopic dermatitis are negatively correlated.^54^^,^^57^ The odds ratio of breastfeeding protecting against asthma was found to be between 0.7 and 0.88.^54^^,^^58^^,^^59^ However, these studies were very heterogeneous in study design and outcome definition. For example, no distinction was made between allergic and non-allergic asthma and different breastfeeding definitions were used. Data from a recent birth cohort in France, examining 1603 children of different age from birth up to 8 years, suggests that breastfeeding has a protective effect against gastrointestinal infections in early infancy and bronchitis/bronchiolitis in the first 2 years of life.^60^ For asthma, only a statistical trend was observed in relation to breastfeeding.^60^

In conclusion, due to a high heterogeneity of the results, meta-analyses provide no convincing data on breastfeeding and its allergy-preventive effects, yet.^45^^,^^61^ Besides the differences in the considered allergic outcomes, main reasons for this high heterogeneity are variations in breastfeeding habits, a lack of human milk immune composition analyses (bioactive and immunomodulatory parameters), the infant's response to human milk (including eg, gut microbiome or thymus size), maternal factors such as the diet, exposure to allergens or microbiota, as well as epigenetic mechanisms. Methodological problems like confounders, interactions, and the absence of standardized methods for assessment limit the conclusions that can be drawn from the existing data. Further studies showing the preventative effect of breastfeeding for allergies as well, are eagerly awaited. These will be important to unravel the components and mechanisms of human milk in allergy prevention and tolerance induction that have been shown in some studies already.

#### Tolerance induction via human milk

There are several studies on the allergy-preventive mechanisms of human milk. The potential allergy-preventive effect discussed so far might stem from an immune system modulation by reducing cytokines and stimulating regulatory T cells, as well as by modulating the infant's intestinal microbiota.

Molecules with immunomodulatory properties in human milk may affect oral tolerance induction and gut microbiota modulation, and consequently the maturation of the immune system. Data from murine models show that mice exposed to egg (ovalbumin) as an aeroallergen were protected against egg allergy when allergens were transferred from the mother to her mice pups via lactation. This breast milk-mediated transfer of antigens to the neonate mouse results in oral tolerance with antigen-specific protection against allergic airway diseases.^62^ Similar animal models have been conducted with house dust mite antigen. However, they showed that early exposure to house dust mite allergens via breast milk increases the allergic sensitization.^63^ This was confirmed in a human epidemiological study, within a subgroup of the EDEN birth cohort from Nancy and Poitiers, recruited from the general population.^64^ In this study, a high content of house dust mite antigen (Der p1) was found in colostrum and transitional breast milk, respectively. The results remained unchanged after checking for potential confounders and were more pronounced in children with allergic mothers.

Breast milk can contribute to the induction of tolerance through several mechanisms. While the consumption of breast milk might protect against egg allergy, it has been found to increase the risk of being exposed to allergens causing, for example, house dust mite allergy. Recent data highlighted an unpredicted potential risk factor for the development of food allergies, emanating from house dust mite allergen in breast milk, which disrupts gut immune homeostasis and prevents oral tolerance induction to bystander food antigens through their protease activity.^63^

Several recent studies point towards a protective effect of the microbiota in allergy prevention.^65^ Exposomal factors such as a western diet, cesarean section, as well as the increased use of antibiotics have a huge impact on the gut microbiota.^66^ A western diet, for example, which is characterized by a high intake of processed foods that are high in fats and sugars, is related to a disturbance of the homeostatic balance of the gut microbiota. These and other early-life factors during the prenatal and the postnatal period temporarily affect the infant gut microbiota and therefore immune system maturation and allergy development.^67^

### Exposure to farm environments protects from allergies

Several epidemiologic studies have assessed the influence of nutritional and non-nutritional exposures on the prevention of allergy development. A recurring and study-site-independent observation is the protective effect of early exposure to farm environments. This farm effect might stem from mechanisms involving the gut as well as nasal microbiota, along with innate and adaptive immune priming. This means that both nutritional and non-nutritional factors might contribute to this effect, as will be further discussed below.

Contact with farm animals in early life seems to protect against allergy development. This farm effect was first observed in the allergy and endotoxin (ALEX) cohort. This cross-sectional study included more than 900 school-aged farm and non-farm children from Switzerland, Germany, and Austria. Riedler et al showed a reduction in prevalence of hay fever, asthma, and atopy of approximately 50% in children with farm exposure.^68^ Based on these data, the hypothesis of a farm effect as protection against allergy was postulated and described in different epidemiologic and prospective studies.^69^

Most of the studies investigated the effects of children growing up on farms in Central Europe (Germany, Austria, and Switzerland). Holbreich et al, however, compared these effects to Amish children living in a traditional community in Indiana (United States). Here, an even greater reduction in the prevalence of asthma, hay fever, and atopy was observed. In accordance with this, the traditional Amish lifestyle and the higher exposure to livestock appears to increase the protective effect even more.^70^ Dissimilarities between communities with different traditional farming lifestyles were observed. While the Amish live on single-family dairy farms, the Hutterites live on large communal farms and are not necessarily exposed to dairy livestock. Stein et al concluded that close contact, especially to cow sheds, might be an important protective factor, as the allergy rate among Amish children was significantly lower than in children living on Hutterite farms.^71^

There are different aspects of the farm effect that might be causative or merely related to the protection. Different independent groups showed that some of these aspects are truly causal, as they could be transferred to animal models of allergic diseases. The hereby observed mechanisms differ in the route of contact (eg, oral, nasal, skin) or time window in which this exposure had a protective effect.^69^ The compounds of interest — for example, bacteria; extracts of mattress dust including cow shed dust — were administered intranasally to mice during or before allergen sensitization. Mattress dust obtained from the farm homes led to a significant reduction in allergen sensitization. By transferring these dust samples to a murine allergy model, the discrepancy of allergy prevalence in Amish as compared to Hutterite cohorts could be simulated.^71^

Nutritional and non-nutritional factors contribute to the reduced prevalence of asthma and allergic disease in farm children. Contact with livestock and consumption of raw cow's milk were the 2 most important factors. Individually, these 2 factors were each able to reduce the risk of asthma and atopic diseases by 50%. The combination of the 2 reduced the risk even further.^68^ Therefore, according to the data of Riedler et al, a combination of exposure via ingestion and inhalation might be a strategy for atopic disease prevention.

The protective effect of consuming raw cow's milk was addressed in more detail in the prospective birth cohort study PASTURE (Protection against Allergy: Study in Rural Environments). This international prospective birth cohort study followed 1133 farm and non-farm children from pregnancy until the age of 6 years. The study results show that consumption of raw cow's milk in early life reduces the risk of rhinitis, otitis, respiratory tract infections, and fever. The effect was lost — especially for rhinitis — when the farm milk was boiled, or when pasteurized or ultra-high-temperature-treated milk was consumed.^72^

Raw cow's milk can contain pathogenic bacteria, which is why the European Food Safety Authority (EFSA) strongly advises against the consumption of raw cow's milk by neonates, infants, and at-risk individuals.^73^

However, a recent meta-analysis on the consumption of raw cow's milk finds protective effects for early asthma, wheezing, hay fever, or allergic rhinitis and atopic sensitization.^74^ The effect could also be found in non-farm children. Nevertheless, the authors also address the small risk of life-threatening infections caused by pathogenic bacteria in raw milk, which is why they explicitly discourage the consumption of raw milk.

Consequently, identifying the protective factors in raw cow's milk is essential for the development of preventative and at the same time harmless nutritional strategies. During milk processing (eg, heat treatment), whey components as well as microorganisms are denaturated. During the homogenization process, the fat globules are reduced in size and uniformly dispersed to prevent phase separation. As a result, heat-sensitive whey proteins (eg, alpha-lactalbumin or beta-lactoglobulin) and shear force-sensitive fat components (eg, omega-3 fatty acids) found in raw milk might lose their protective effect.^75^

In addition, the variety of food introduced during the first year of life was inversely associated with the risk of developing atopic dermatitis, as was recently reported within the prospective birth cohort study PASTURE.^76^ The study demonstrated that the consumption of yogurt during the first year of life had a protective effect, suggesting the presence of protective microbial components in fermented milk products. The protection was linked to increased levels of short chain fatty acids (SCFA) propionate and especially butyrate in the gut. This consequently suggests that the consumption of foods rich in microorganisms, SCFAs or fiber for intestinal microbial generation of these SCFAs might have a protective effect.^77^

Apart from microbial components entering via the oral route, the inhalation of microbial components present in the farm air might also provide protection against allergies. The effects of farm microbe exposure were assessed in several studies by measuring bacterial and fungal markers in mattress or room dust. In general, farm children were found to be exposed to a greater variety of environmental microorganisms than non-farm children. In this context, a higher diversity in both the mattress dust microbiota and the children's nasal microbiota was found to be protective.^78^^,^^79^ A recent publication showed that the protective effect against asthma development was independent of the bacterial richness in the house dust microbiota, but rather related to reduced proinflammatory immune responses to specific bacterial cell wall components. These findings indicate that a farm-like house dust microbiota might improve tolerance mechanisms toward microbial exposures.^80^

Exposure to a farm environment during an early developmental age seems to be crucial for achieving the observed protective effect. Especially the first year of life was found to be a key time frame in the prevention of allergic responses.^68^

## Conclusion

Many factors linked to the western lifestyle have been associated with the development of allergic diseases. The challenge in the forthcoming years is to identify factors that can be targeted to prevent and reduce allergies.

The farm effect exemplifies how important exposomal factors are in allergy prevention. Since most of the exposure factors are modifiable, the studies summarized here open up perspectives on the prevention of allergic diseases. Additional studies proving the preventative effect of breastfeeding for allergies, are eagerly awaited. These will be central to unravel the components and mechanisms of human milk in allergy prevention and tolerance induction. Moreover, we need further research on the plethora of protective components in human milk, as they will be an important strategy to unravel new preventative compounds for administration in this important window of opportunity.

## Abbreviations

IL, interleukins; Ig, immunoglobilin; GALT, gut associated lymphoid tissue; OVA, ovalbumin; SCFA, short-chain fatty acids; TGF-β, transforming growth factor-beta.

## Author contribution

IAM initiated the concept.

MF made the first draft.

The other authors participated to the development of the document.

All authors reviewed and approved the final manuscript.

## Ethics approval and consent to participate

Not applicable; the manuscript does not report on or involve the use of any animal or human data or tissue.

## Consent for publication

All authors agreed to the publication of this work.

## Availability of data and materials

This is a review article.

## Funding

This review article was funded by HiPP GmbH & Co. Vertrieb KG, Pfaffenhofen, Germany.

## Data availability statement

The data that support the findings of this study are freely available in Pubmed at https://pubmed.ncbi.nlm.nih.gov/, in the University of Nebraska Food Allergy Research and Resource Program, available at https://farrp.unl.edu, and in the 10.13039/100000092US National Library of Medicine Clinical Trials register, available at https://clinicaltrials.gov.

## Declaration of competing interest

With the exception of the financial support for workshop participation and manuscript preparation by HiPP GmbH & Co. Vertrieb KG, Pfaffenhofen, Germany, the authors report no conflicts of interest.
